# Enhancing Cognitive Inclusion at Work: Empathy, Technology, and Resilience for Employees with Cognitive Impairments

**DOI:** 10.1002/alz70858_107529

**Published:** 2025-12-26

**Authors:** Josephine McMurray, AnneMarie Levy, Sabah Rasheed, Ashley Cole, Kristina Marie Kokorelias, Jennifer Boger, Catherine Burns, Jim Mann, Arlene Astell

**Affiliations:** ^1^ Wilfred Laurier University, Waterloo, ON, Canada; ^2^ Wilfrid Laurier University, Waterloo, ON, Canada; ^3^ Wilfrid Laurier University, Brantford, ON, Canada; ^4^ Univeristy of Guelph, Guelph, ON, Canada; ^5^ Sinai Health, Toront, ON, Canada; ^6^ University of British Columbia Okanagan, Kelowna, BC, Canada; ^7^ Waterloo University, Waterloo, ON, Canada; ^8^ Alzheimer Advocate, Vancouver, BC, Canada; ^9^ University of Toronto, Toronto, ON, Canada

## Abstract

**Background:**

The global workforce is aging ^1^, increasing the prevalence of Mild Cognitive Impairment (MCI) and Young‐Onset Dementia (YOD)^2^, and driving organizations to adopt more comprehensive approaches to inclusion^3^. These conditions pose unique challenges for employers balancing inclusivity with operational efficiency. Traditional accommodation approaches often prove inadequate, relying on outdated practices unsuited to the evolving needs of employees with cognitive impairments. This study examines the intersection of empathetic organizational practices, technology integration, and resilience‐building strategies to support workers with cognitive disabilities.

**Method:**

Using a multi‐level comparative case study design, we conducted 97 semi‐structured interviews in two diverse Canadian organizations—one in the public sector and one in healthcare. Drawing on a socio‐technical systems framework and the Job Demands‐Resources (JD‐R) model, we explored how these organizations manage job demands and resources for employees with MCI and YOD. Interviews addressed workplace culture, accommodation practices, managerial support, and technology's role in creating inclusive environments.

**Result:**

Effective accommodations combined empathetic leadership, flexible management, and the strategic deployment of both digital and non‐digital technologies. Organizations enabling adaptive decision‐making and iterative feedback loops demonstrated greater resilience. However, technology alone was insufficient; a person‐centered, adaptive approach aligned with organizational workflows was vital to reduce strain and enhance job performance.

**Conclusion:**

Findings underscore the need for empathetic, flexible workplaces where managerial and technological systems respond to employees’ evolving cognitive needs. A conceptual model of “strain and resilience cycles” highlights how organizational structures and technology must continuously adapt for sustained support. The results emphasize fostering an inclusive culture, reducing stigma, and leveraging technology in ways that enhance—rather than hinder—employees’ experiences. By integrating empathetic leadership, personalized accommodations, and adaptive feedback systems, organizations can improve both operational efficiency and the well‐being of employees with cognitive impairments.

